# Colitis Is Associated with Loss of the Histidine Phosphatase LHPP and Upregulation of Histidine Phosphorylation in Intestinal Epithelial Cells

**DOI:** 10.3390/biomedicines11082158

**Published:** 2023-08-01

**Authors:** Markus Linder, Dritan Liko, Venkatesh Kancherla, Salvatore Piscuoglio, Michael N. Hall

**Affiliations:** 1 Biozentrum, University of Basel, 4056 Basel, Switzerland; 2 Institute of Medical Genetics and Pathology, University Hospital Basel, 4031 Basel, Switzerland; 3 Visceral Surgery and Precision Medicine Research Laboratory, Department of Biomedicine, University of Basel, 4031 Basel, Switzerland

**Keywords:** histidine phosphorylation, LHPP, inflammatory bowel disease, colitis

## Abstract

Protein histidine phosphorylation (pHis) is a posttranslational modification involved in cell cycle regulation, ion channel activity and phagocytosis. Using novel monoclonal antibodies to detect pHis, we previously reported that the loss of the histidine phosphatase LHPP (phospholysine phosphohistidine inorganic pyrophosphate phosphatase) results in elevated pHis levels in hepatocellular carcinoma. Here, we show that intestinal inflammation correlates with the loss of LHPP in dextran sulfate sodium (DSS)-treated mice and in inflammatory bowel disease (IBD) patients. Increased histidine phosphorylation was observed in intestinal epithelial cells (IECs), as determined by pHis immunofluorescence staining of colon samples from a colitis mouse model. However, the ablation of *Lhpp* did not cause increased pHis or promote intestinal inflammation under physiological conditions or after DSS treatment. Our observations suggest that increased histidine phosphorylation plays a role in colitis, but the loss of LHPP is not sufficient to increase pHis or to cause inflammation in the intestine.

## 1. Introduction

Protein histidine phosphorylation, a poorly characterized posttranslational modification, refers to the addition of a phosphate group to the imidazole ring of histidine via a heat and acid labile phosphoramidate (P-N) bond. Both nitrogens in the histidine imidazole ring can be phosphorylated, resulting in the formation of two isomers: 1-phosphohistidine (1-pHis) and 3-phosphohistidine (3-pHis). So far, three mammalian histidine phosphatases (LHPP, PGAM5 and PHPT1) and two histidine kinases (NME1, NME2) have been described [1,2].

We previously reported that murine and human hepatocellular carcinomas (HCCs) exhibit elevated histidine phosphorylation and decreased levels of the histidine phosphatase LHPP. The expression of LHPP correlated with HCC patients’ survival, indicating that LHPP has a tumor-suppressive function. Reintroduction of LHPP resulted in decreased pHis levels in vitro and prevented tumor formation in an HCC mouse model, suggesting that elevated pHis is pathological [3]. Recent publications provided additional evidence that LHPP acts as a tumor suppressor in other cancers, including pancreatic cancer [4], glioblastoma [5] and oral squamous cell carcinoma [6]. Importantly, the expression of LHPP protein was shown to correlate with survival of patients suffering from colorectal cancer (CRC) [7], a type of cancer that is often linked to chronic inflammation [8]. However, the role of LHPP in inflammation and/or chronic inflammatory disease has not yet been addressed.

Inflammatory bowel disease (IBD) is a major risk factor for CRC [9]. IBD is a general term for intestinal disorders characterized by chronic colitis, such as Crohn’s disease (CD) and ulcerative colitis (UC). The etiology and the molecular pathophysiology of IBDs are incompletely understood, resulting in insufficient progress in the development of novel therapies [10]. Importantly, inhibition or deletion of the K+ channel KCa3.1 prevents the progression of colitis [11], and PGAM5 inhibits KCa3.1 by dephosphorylating NME2 at pHis118 [12]. Taken together, the points above suggest that pHis may play a role in the pathology of IBD.

Here, we aimed to investigate whether histidine phosphorylation is linked to IBD by analyzing the expression levels of known histidine phosphatases and kinases in colon samples from CD and UC patients and from a mouse model of colitis. We analyzed histidine phosphorylation in DSS-driven inflamed mouse colon tissue samples and generated LHPP knockout mice to study the effect of the loss of this histidine phosphatase on colitis development and histidine phosphorylation in vivo.

## 2. Materials and Methods

### 2.1. Mice 

To generate *Lhpp*^−/−^ mice, Exon 2 of the mouse *Lhpp* gene was deleted using CRISPR/Cas9-mediated non-homologous end joining (NHEJ). Two gRNAs were designed to target the *Lhpp* Introns 1 (IVS1) and 2 (IVS2). The sequences targeting the respective Introns IVS1-CATCTGACTCACATCATGTGAGG and IVS2-GCATCCTGAAGCTAGCCTTGAGG were selected for optimal on-target activity using the CRISPOR online tool [13]. NHEJ events at the gRNA target sites led to the excision of the genomic fragment containing Exon 2, resulting in a *Lhpp*-null allele. CRISPR/Cas9-mediated modification of the *Lhpp* sequence was carried out by electroporation of fertilized mouse oocytes as previously described [14]. The *Lhpp*^−/−^ and *Lhpp*^+/+^ mice were maintained in a C57BL/6J genetic background. C57BL/6J wild-type mice were purchased from Janvier Labs. The number of animals used for the individual experiments is stated in the figure legends. Experimental colitis was induced in 8- to 12-week-old male mice by administering 2.5% DSS in drinking water for up to 7 days according to published protocols [15]. All animal experiments conducted were compliant with federal laws and guidelines, and were approved by the veterinary office of Basel-Stadt (approval number: 3022; approval date: 3 October 2019).

### 2.2. Histology 

Immunohistochemistry (IHC) and H&E staining was performed as previously described [16]. The following primary antibodies were used: cleaved Caspase 3 (9664; CST, Danvers, MA, USA), Ki-67 (12202; CST, Danvers, MA, USA), and LHPP (NBP1-83272; Novus Biologicals, Centennial, CO, USA). For immunofluorescence (IF) staining, the colons were flushed with ice-cold PBS (pH 8.5), cryo-fixed in a cryo-embedding matrix at optimal cutting temperature (OCT) and stored at −80 °C. To prevent heat- and acid-mediated pHis degradation, all steps during the IF staining process were performed at 4 °C, and the pH of all buffers was adjusted to 8.5. After cutting, the colon cryo-sections (10 μm) were fixed for 1 h in 4% PFA, washed with 1× PBS, blocked for 1 h using a blocking buffer (1× PBS, 1% BSA, 0.05% Triton-X 100) and subsequently incubated O/N with the primary antibodies (3-pHis: rabbit, SC44-1; F4/80: rat, ab6640; Abcam, Cambridge, UK) diluted in a blocking buffer. Afterwards, the slides were rinsed with PBS and incubated for 1 h with secondary antibodies (Alexa Fluor 488 anti-rabbit and Alexa Fluor 568 anti-rat; Invitrogen, Waltham, MA, USA) and DAPI (4083; CST, Danvers, MA, USA). Finally, the stained sections were washed and mounted with a water-based mounting medium (H-1400; Vector Laboratories, Newark, CA, USA). 

### 2.3. Immunoblotting 

Immunoblots were performed and quantified as previously described [3]. To detect pHis, the following monoclonal primary antibodies were used: 1-pHis (0.5 μg/mL, SC1-1) and 3-pHis (0.5 μg/mL, SC44-1). For regular immunoblot analysis, the following primary antibodies were used: calnexin (ADI-SPA-860; Enzo Life Sciences, Farmingdale, NY, USA), LHPP (15759-1-AP; Proteintech, Rosemont, IL, USA), NME1 (3345; CST, Danvers, MA, USA), NME2 (ab60602; Abcam), PGAM5 (ab126534; Abcam, Cambridge, UK) and PHPT1 (LS-C192376; LSBio, Seattle, WA, USA). 

### 2.4. Analysis of the Publicly Available Transcriptomic Dataset 

For mRNA expression analysis, the Affymetrix GeneChip Human Genome U133 Plus 2.0 gene expression dataset E-GSE16879 was downloaded from ArrayExpress. The dataset included mRNA expression data from colon biopsies derived from 6 healthy donors and 43 IBD patients (24 UC, 19 CD) from a previously published study [17]. Detailed information regarding this publicly available dataset and the corresponding ethical committee approval can be found in the original publication by Arijs et al. [17]. The probes were matched with the gene names by using biomaRt R package. Afterwards, the gene expression levels of *LHPP*, *PGAM5*, *PHPT1*, *NME1* and *NME2* were analyzed for different conditions (CTRL, UC and, CD).

### 2.5. Statistical Analysis 

Data analysis was performed with PRISM 8.0 (GraphPad, Boston, MA, USA). Single comparisons were performed by an unpaired 2-tailed Student’s *t*-test. Comparisons of multiple groups were performed by one-way ANOVA, followed by Tukey’s post hoc test for multiple comparison. Data are shown as the mean ± SEM; *, *p* < 0.05; **, *p* < 0.01; ***, *p* < 0.001; ****, *p* < 0.001.

## 3. Results

**Figure 1:**
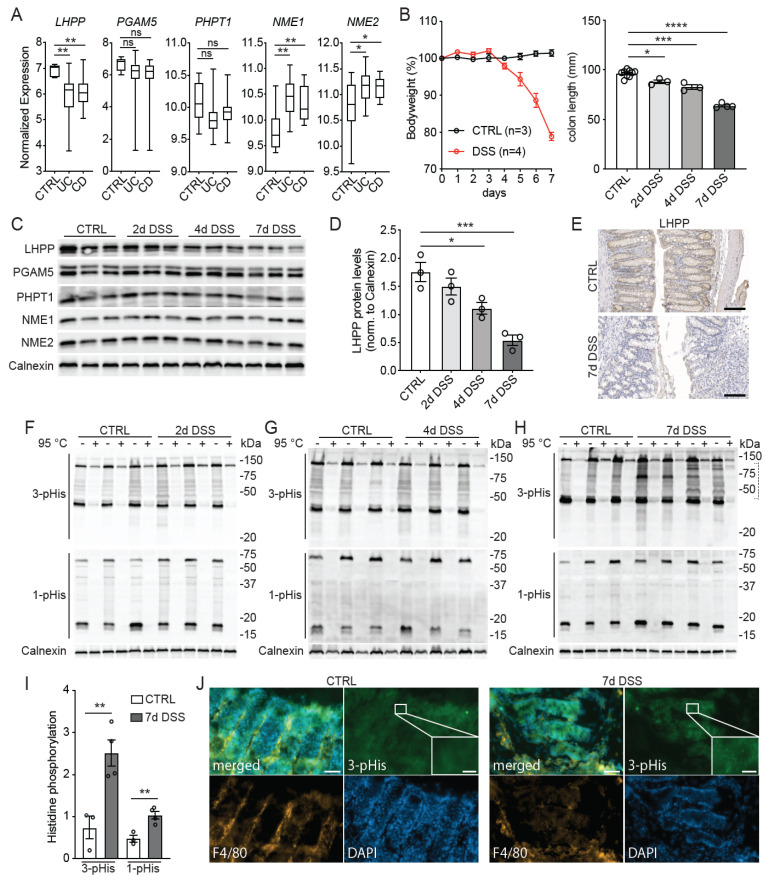
Intestinal inflammation correlates with downregulation of LHPP and increased histidine phosphorylation. (**A**) Analysis of a publicly available dataset comparing the mRNA expression of known histidine phosphatases (LHPP, PGAM5, PHPT1) and histidine kinases (NME1, NME2) in colon tissue from healthy patients (CTRL) with that of patients suffering from ulcerative colitis (UC) and Crohn’s disease (CD). (**B**) Bodyweight and colon length of mice treated (DSS, *n* = 4) and untreated (CTRL, *n* = 3) with DSS at the indicated timepoints (days). (**C**) Immunoblot analysis of histidine phosphatases and kinases in colon lysates of DSS-treated and untreated mice at the indicated timepoints (*n* = 3). (**D**) Quantification of LHPP protein levels normalized to calnexin at different timepoints of the DSS treatment (*n* = 3). (**E**) Immunohistochemistry visualization of LHPP in the colon samples of mice treated (DSS) and untreated (CTRL) for 7 days with DSS. Scale bar: 100 μm. (**F**–**H**) Immunoblot analysis of 1- and 3-pHis levels in colon samples from DSS-treated (DSS, *n* = 3−4) and untreated (CTRL, *n*= 3) mice at the indicated timepoints. (**I**) Quantification of the 1− and 3−pHis levels in (**H**). (**J**) DAPI and immunofluorescence staining of colon samples from untreated mice (CTRL) and mice treated with DSS for 7 days. Scale bars: 50 μm (low magnification) and 10 μm (high magnification). Data are shown as the mean ± SEM; *, *p* < 0.05; **, *p* < 0.01; ***, *p* < 0.001; ****, *p* < 0.001; ns = not significant.

To investigate if histidine phosphorylation plays a role in colitis, we analyzed a publicly available transcriptomic profile (E-GEOD-16879) from colon samples of healthy humans and treatment-naïve IBD patients [17]. Patients suffering from CD or UC showed significantly decreased expression of *LHPP*, but no difference in the expression of the other two known histidine phosphatase genes *PGAM5* and *PHPT1* (Figure 1A). The RNA expression levels of both histidine kinases *NME1* and *NME2* were significantly upregulated in IBD patients (Figure 1A). On the basis of these findings, we hypothesized that elevated pHis, via downregulation of the histidine phosphatase LHPP and upregulation of the histidine kinases NME1 and NME2, contributes to disease progression in IBD patients.

To examine the role of pHis in IBD further, we induced experimental colitis by treating wild-type mice with dextran sodium sulfate (DSS) [15]. Mice exhibited mild colitis-like symptoms after 2 to 4 days, which developed into severe colitis with a bodyweight loss of up to 20% and a significantly decreased colon length, as observed after one week of treatment (Figure 1B), which was in line with previously published reports using this model [18,19]. Next, we analyzed the protein expression of known histidine phosphatases and kinases at different timepoints of the DSS treatment. The histidine phosphatase LHPP was significantly downregulated in the colon of wild-type mice after 4 days of DSS treatment, as determined by immunoblotting (Figure 1C,D). After one week of treatment, LHPP expression was further reduced, indicating that LHPP expression was negatively correlated with the severity of colitis (Figure 1C,D). Importantly, immunohistochemistry (IHC) staining confirmed this observation and further revealed that the DSS-dependent downregulation of LHPP was mainly due to reduced expression in the intestinal epithelial cells rather than in the infiltrating immune cells (Figure 1E). In line with the results from human IBD patients shown in Figure 1A, the expression of other known histidine phosphatases (PGAM5 and PHPT1) remained unchanged in DSS-treated mice (Figure 1C). However, contrary to the human RNA expression data, the protein levels of both histidine kinases NME1 and NME2 were also unchanged in the colons of DSS-treated mice (Figure 1C). 

We next analyzed histidine phosphorylation in colon samples isolated at different timepoints of the DSS treatment. Immunoblot analysis of the colon lysates showed significantly upregulated 1- and 3-pHis levels after 7 days of DSS treatment, but not at earlier timepoints (Figure 1F–I). In agreement with the IHC results described above, DSS treatment increased pHis exclusively in IECs, not in the infiltrating macrophages (F4/80-positive cells), as shown by immunofluorescence staining (Figure 1J). As we observed significant downregulation of LHPP at 4 days of DSS treatment but changes in pHis only after 7 days (i.e., the loss of LHPP preceded increased pHis), we speculate that the loss of LHPP contributes to high levels of pHis and disease progression. 

**Figure 2:**
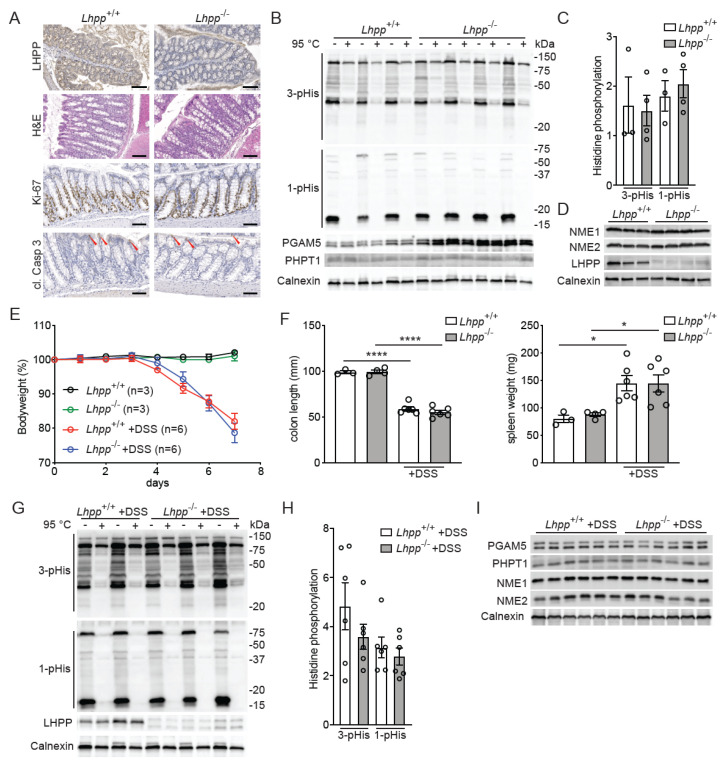
LHPP is dispensable for the development of colitis. (**A**) IHC (LHPP, Ki67, cleaved caspase 3) and H&E staining of colon samples of 1.5-year-old *Lhpp*^+/+^ and *Lhpp*^−/−^ mice. Red arrowheads indicate cleaved (cl.) caspase 3−positive cells. Scale bars: 100 μm. (**B**) Immunoblot analysis of 1− and 3−pHis and histidine phosphatase protein levels in colon samples from *Lhpp*^+/+^ (*n* = 3) and *Lhpp*^−/−^ (*n* = 4) mice. (**C**) Quantification of the 1− and 3−pHis levels in B. (**D**) Immunoblot analysis of NME1, NME2 and LHPP in colon samples from *Lhpp*^+/+^ (*n* = 3) and *Lhpp*^−/−^ (*n* = 4) mice. (**E**) Bodyweight during DSS treatment (*n* = 3 for untreated animals; *n* = 6 for DSS-treated animals). (**F**) Colon length and spleen weight of the control animals (*n* = 3) or after 7 days DSS treatment (*n* = 6). (**G**) Immunoblot analysis of 1− and 3−pHis and LHPP protein levels in colon samples from *Lhpp*^+/+^ (*n* = 2) and *Lhpp*^−/−^ (*n* = 3) mice treated with DSS for 7 days. (**H**) Quantification of the 1− and 3−pHis levels in (**G**) with *n* = 6. (**I**) Immunoblot analysis of histidine phosphatases and kinases in the colon samples from *Lhpp*^+/+^ and *Lhpp*^−/−^ mice treated with DSS for 7 days (*n* = 6). Data are shown as the mean ± SEM; *, *p* < 0.05; ****, *p* < 0.001.

To obtain insights into the role of LHPP in normal development and in the progression of colitis, we generated full-body LHPP knockout (*Lhpp*^−/−^) mice using CRISPR/Cas9. The *Lhpp*^−/−^ mice were vital, fertile and indistinguishable from their littermate controls. Histological analysis of the colonic mucosa did not reveal significant differences between the *Lhpp*^−/−^ and *Lhpp*^+/+^ littermates (Figure 2A). Colons of 1.5-year-old *Lhpp*^−/−^ mice clearly displayed the deletion of LHPP, as shown by LHPP IHC staining, but no signs of cancer and/or inflammation (Figure 2A). In line with the normal colonic crypt morphology, the intestinal epithelium of *Lhpp*^−/−^ mice displayed no differences in proliferation or apoptosis, as shown by Ki-67 and cleaved caspase 3 IHC staining (Figure 2A). Moreover, immunoblot analysis revealed no significant difference in the levels of 1- or 3-pHis in the large intestine lysates of *Lhpp*^−/−^ and *Lhpp*^+/+^ littermates (Figure 2B,C). Colons from *Lhpp*^−/−^ mice did not display any differences in histidine phosphatase PHPT1 protein levels but exhibited elevated PGAM5 expression, possibly as a compensatory mechanism for the loss of LHPP (Figure 2B). Importantly, loss of LHPP did not affect the two histidine kinases NME1 and NME2, as their protein levels in colons of *Lhpp*^−/−^ mice remained unchanged (Figure 2D). 

On the basis of our observation that DSS treatment resulted in the loss of LHPP in wild-type mice (Figure 1C–E), we formulated the hypothesis that LHPP plays an essential role during colitis development. To determine whether loss of LHPP indeed affects the progression of colitis, we treated *Lhpp*^−/−^ and *Lhpp*^+/+^ littermates with DSS. We did not detect any significant difference in the bodyweight loss after DSS treatment and no difference in the time to the onset of colitis, which was at 4–5 days after the start of treatment for both genotypes (Figure 2E). Other colitis-related parameters such as colon shrinkage and elevated spleen weight were also similar in *Lhpp*^−/−^ and *Lhpp*^+/+^ mice (Figure 2F), indicating that the loss of LHPP does not impact the development of colitis. Finally, we did not detect genotype-dependent changes in intestinal 1- and 3-pHis levels after DSS treatment (Figure 2G,H) or differences in expression of the two histidine phosphatases PGAM5 and PHPT1, or the two histidine kinases NME1 and NME2 (Figure 2I).

## 4. Discussion

In summary, we show that the loss of LHPP and increased histidine phosphorylation in intestinal epithelial cells correlate with colitis. However, the loss of LHPP does not appear to be sufficient for either the observed increase in pHis or inflammation, at least in mice. Increased *NME1* and *NME2* expression or activity, which we observed in IBD patients but not in *Lhpp*^−/−^ mice, might be required in addition to the loss of LHPP. 

The role of LHPP and/or histidine phosphorylation in IBD has not yet been investigated; however, IBD patients have a significantly elevated risk of developing colorectal cancer (CRC) [20,21], and several studies have provided evidence that LHPP is lost in CRCs and that the overexpression of LHPP can suppress the development of CRC in vivo [7,22,23]. A previous publication showed that a high-fat diet + azoxymethane (AOM)/DSS-induced colorectal adenomas that displayed downregulation of the mRNA expression of *Lhpp* [24]. Importantly, when these mice were treated with the traditional Chinese medicine compound “Canmei formula”, the low LHPP levels could be restored, and the development of adenoma was significantly reduced [24]. Future studies are necessary to evaluate if the reintroduction of LHPP can also prevent or rescue colitis, and whether targeting histidine phosphorylation could be a path forward to develop novel therapeutics in the field of IBD and/or CRC.

Little is known about the role of pHis in inflammation. Fuhs et al. reported that both malignant epithelial cells and macrophages show high pHis levels in vitro, and suggested that pHis is important in phagocytosis [2]. As activated macrophages are key players in colitis [25], we originally expected that the high pHis levels we observed in our experimental system would be in the immune cells. However, the results from our IF staining indicated that DSS treatment triggers pHis in the IECs. As we observed high pHis levels only in late-stage colitis, we cannot exclude the possibility that IECs upregulate pHis as a response to infiltrating immune cells. Alternatively, the upregulated pHis levels in epithelial cells might promote inflammation by inducing the production of pro-inflammatory signaling molecules and the recruitment of macrophages. It remains to be determined whether increased pHis is a consequence or a cause of inflammation in IBD. 

It is also necessary to further investigate the role of pHis in the complex interplay between IECs and immune cells. Finally, it will be important to identify histidine phosphorylated proteins and to determine their role in inflammatory diseases.

## 5. Conclusions

The histidine phosphatase LHPP is downregulated in colon samples from Crohn’s disease and ulcerative colitis patients, and from mice with experimentally induced colitis, suggesting the involvement of this protein in IBD. However, the deletion of LHPP alone was not sufficient to induce inflammation in the colon or to promote the DSS-induced colitis phenotype in mice. Further studies are needed to dissect the role of LHPP and histidine phosphorylation in IBD.

## Data Availability

The data presented in this study are available on request from the corresponding author.
